# The possible involvement of circRNA DMNT1/p53/JAK/STAT in gestational diabetes mellitus and preeclampsia

**DOI:** 10.1038/s41420-022-00913-w

**Published:** 2022-03-16

**Authors:** Dongqin Bao, Chaohui Zhuang, Yan Jiao, Li Yang

**Affiliations:** Center for Reproductive Medicine, The Affiliated Shuyang Hospital of Xuzhou Medical University, Suqian City, Jiangsu Province China

**Keywords:** Cell biology, Diseases

## Abstract

Circular RNA (circRNA) plays an important role in biological processes of gestational diabetes mellitus (GDM) and preeclampsia (PE). However, the mechanisms for circRNA DMNT1 (circ-DMNT1) in GDM and PE remain unclarified. The expression levels of circ-DMNT1 and p53 in GDM and PE were quantified by quantitative real-time polymerase chain reaction (qRT-PCR) and western blot analysis. When the expression of circ-DMNT1 or p53 was abnormal, cell counting kit-8 (CCK-8) assay, bromodeoxyuridine (BrdU) staining, flow cytometry, cell scratch, and Transwell assays were used to assess cell viability, proliferation, cell cycle, apoptosis, migration, and invasion of trophoblast cells, respectively. Subsequently, the binding relationship between circ-DMNT1 and p53 was verified by RNA pull-down and RIP analysis, followed by the determination of JAK/STAT pathway-related protein expression levels using western blot analysis. Both circ-DMNT1 and p53 were highly expressed in GDM and PE. Upregulation of circ-DMNT1 or p53 inhibited trophoblast cell viability, proliferation, migration, and invasion, meanwhile promoting cell apoptosis but blocking cell cycle progression. However, downregulation of circ-DMNT1 or p53 induced trophoblast cell survival. In GDM and PE, circ-DMNT1 activated the JAK/STAT pathway by binding to p53, which resulted in increased expression levels of p-JAK and p-STAT. The results suggested that circ-DMNT1 was involved in the deterioration of GDM and PE, possibly through inducing p53 expression and activating the JAK/STAT signaling pathway.

## Introduction

Hypertensive disorders such as preeclampsia (PE) and metabolic diseases such as gestational diabetes mellitus (GDM) are pregnancy-specific complications [1]. It is reported that abnormal development of the placenta is the main cause of PE pathology, and the incidence of PE in China is 9.4–10.4% [2]. In addition, GDM is often accompanied by vascular lesions and placental dysfunction is the common pregnancy complication in China with an incidence of 7.61% [3]. Studies have found that extra-villous trophoblast (EVT) is critical for the development of the placenta, and its dysfunctions increase the harmful effects of PE and GDM, which will have negative impacts on the developing fetus, increasing the risk of obesity and neuropsychological disorders in offspring [4, 5]. Currently, diet modification, physical activity, and drug therapy are the primary approaches to reducing fetal concomitant diseases and improving pregnancy outcomes [6]. However, optimal strategies for preventing and treating PE and GDM still need to be developed.

Circular RNAs (circRNAs) are covalently closed single-stranded RNAs with 3’ and 5’ terminal junctions in exons, which are found in several highly conserved species [7]. circRNAs are crucial in biological processes and control *cis*- and/or *trans*-gene expression by participating in several known levels of regulation [8]. Accumulated evidence suggested that the combination of circRNAs might be a superior candidate for both PE or GDM biomarkers and adjuvant therapy strategies [9, 10]. CircRNA DNA methyltransferase 1 (circ-DNMT1), an example of carcinogenic circRNA, increases cell malignant behavior [11]; meanwhile, it has been reported that circ-DNMT1 is involved in the development of breast cancer by promoting cancer cell survival and invasion, as well as inhibiting apoptosis [12]. Although circ-DNMT1 has an important effect on cell biological function, the mechanism of circ-DNMT1 in PE and GDM needs further elucidation.

P53 controls cell division, DNA replication and cell cycle, and plays a critical role in the differentiation of various tissues and organs [13]. Normally, the p53 protein is switched off during cell stress and uncontrolled division and proliferation [14], while when cell damage cannot be repaired, p53 triggers programmed cell death by activating apoptosis-related genes [15]. Previous studies have shown that p53 is implicated in the metastasis of various cancers by modulating the survival and migration potential of cancer cells [16]. Remarkably, EVT cells (EVTs) exhibit a phenotype very similar to that of cancer cells in terms of invasiveness [17]. Additionally, ectopic circ-DNMT1 can interact with p53 to promote its nuclear translocation [11]. Nevertheless, the regulatory mechanism of circ-DNMT1/p53 in EVTs remains unclear.

Additionally, JAK/STAT signaling pathway is a commonly expressed intracellular signal transduction pathway, which is involved in many key biological processes including cell proliferation, differentiation, apoptosis, and immune regulation [18]. Studies have reported that p53 regulates the JAK/STAT signaling pathway through a potential interaction with activated STATs [19]. However, the role of p53-JAK/STAT in PE and GDM remains poorly elucidated.

Therefore, this study aimed to investigate the underlying mechanism for circ-DNMT1 in regulating the biological function of EVTs and clarified that circ-DNMT1 participated in the occurrence and development of PE and GDM by combining p53 and activating the JAK/STAT signaling pathway.

## Results

### circ-DMNT1 was upregulated in PE and GDM placenta and located in the cytoplasm of HTR-8/SVneo cells

**Fig. 1:**
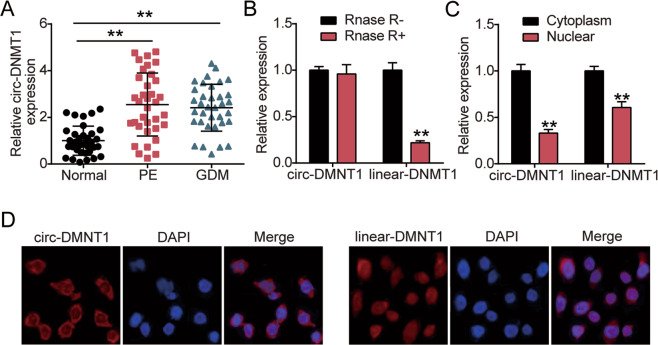
circ-DMNT1 was upregulated in PE and GDM placenta and located in the cytoplasm of HTR-8/SVneo cells. **A** qRT-PCR analysis of circ-DMNT1 expression in PE (*N* = 35) and GDM placentas (*N* = 35). **P < 0.001. **B** The expression of circ-DMNT1 and linear DMNT1 were detected by RT-qPCR in HTR-8/SVneo cells with or without RNase R treatment, **P < 0.001 compared with the RNase R− group. **C** The nuclear and cytoplasmic qRT-PCR was carried out to verify subcellular localization of circ-DMNT1 and linear DMNT1in HTR-8/SVneo cells, **P < 0.001 compared with the cytoplasm. **D** Subcellular localization of circ-DMNT1 and linear DMNT1 was determined by in situ hybridization assay. *N* = 3, repetition = 3.

To explore the role of circ-DMNT1 in PE and GDM, we characterized the trends in the expression of circ-DMNT1 in PE and GDM. The expression of circ-DMNT1 in the placenta of 35 cases with PE and 35 pregnant women with GDM was measured using qRT-PCR. As shown in Fig. 1A, circ-DMNT1 levels were about 2.5 times in PE patients and 2.3 times in GDM patients higher than in healthy pregnant women. Circ-DMNT1 might be related to PE and GDM. The HTR-8/SVneo cell lysate was then treated with RNase R to assess the stability of circ-DMNT1 and linear DMNT1. The results showed that the expression of linear DMNT1 decreased by about 75% after RNase R treatment, while the level of circ-DMNT1 did not change significantly, indicating that circ-DMNT1 had some anti-digestion effect on RNase R and stable circle structure (Fig. 1B). To assess the subcellular localization of circ-DMNT1 and linear DMNT1 in HTR-8/SVneo cells, the expression levels of these two RNAs in the nucleus and cytoplasm were measured. Results from Fig. 1C revealed that circ-DMNT1 and linear DMNT1 were mainly distributed in the cytoplasm. In addition, FISH experiments confirmed the results of subcellular localization (Fig. 1D). Taken together, the data suggested that circ-DMNT1 existed primarily in the cytoplasm of trophoblast cells as a stable circular transcript and was upregulated in the placentas of PE and GDM pregnant women.

### Silencing circ-DMNT1 promoted trophoblast cell proliferation, migration, and invasion, inhibited apoptosis, and induced cell cycle progression

**Fig. 2:**
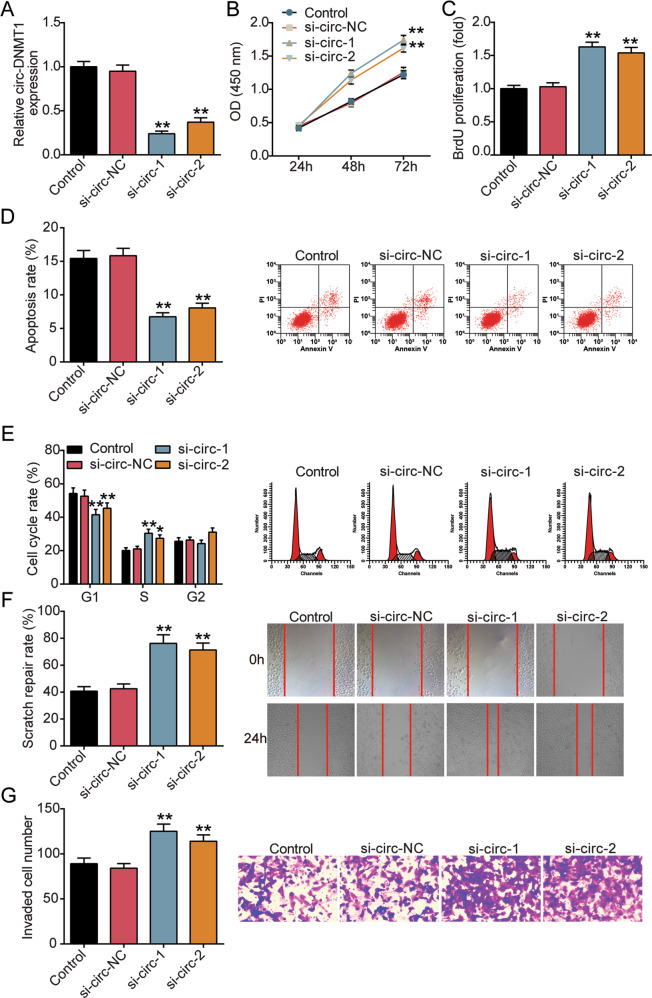
Silencing circ-DMNT1 promoted trophoblast cell proliferation, migration, and invasion, inhibited apoptosis, and induced cell cycle. **A** HTR-8/SVneo cells were transiently transfected with circ-DMNT1 siRNA#1 and #2 for 48 h. qRT-PCR analysis knockdown expression of circ-DMNT1. **B** CCK-8 assays were implemented to detect the viability of si-circ-1/si-circ-2-treated HTR-8/SVneo cells. **C** BrdU assays were implemented to detect the proliferation of si-circ-1/si-circ-2-treated HTR-8/SVneo cells. **D** The apoptotic rates of HTR-8/SVne cells transfected with si-circ-1 or si-circ-2 were measured by flow cytometry. **E** The cell cycle of HTR-8/SVne cells transfected with si-circ-1 or si-circ-2 was measured by flow cytometry. **F** Wound scratch assay was performed to detect the role of circ-DMNT1 on si-circ-1 or si-circ-2-treated HTR-8/SVne cell migration in vitro. **G** Transwell assays were performed to detect the invasion of HTR-8/SVne cells transfected with si-circ-1 or si-circ-2. *P < 0.05, **P < 0.001 compared with si-circ-NC. *N* = 3, repetition = 3.

Afterward, the effects of circ-DMNT1 on the biological function of trophoblast cells were investigated. When siRNA targeting circ-DMNT1 was transfected into HTR-8/SVneo cells, qRT-PCR showed that circ-DMNT1 levels in si-circ-1 and si-circ-2 were reduced by about 80 and 65%, respectively, compared with that in the si-circ-NC group (Fig. 2A). Cell viability and cell proliferation of HTR-8/SVneo cells were analyzed by CCK-8 and BrdU respectively. Data from CCK-8 assay displayed that the cell viability of si-circ-1 and si-circ-2 was upregulated by 1.4- and 1.28-fold, respectively, compared with that in the si-circ-NC group (Fig. 2B). BrdU staining revealed that HTR-8/SVneo cell proliferation was downregulated after interference with circ-DMNT1 (Fig. 2C). In addition, flow cytometry analysis showed that the apoptosis rate in si-circ-1 and si-circ-2 groups were reduced to about 60 and 50% of that in the si-circ-NC group, respectively (Fig. 2D). Moreover, results in Fig. 2E revealed that low circ-DMNT1 reduced the G1 phase ratio and increased the S phase ratio. The wound healing experiment showed that the cell migration in si-circ-1 and si-circ-2 groups increased by about 1.8 and 1.7 times, respectively (Fig. 2F). Similarly, the Transwell assay revealed that circ-DMNT1 silence increased the invasiveness in HTR-8/SVneo cells by about 1.3 times (Fig. 2G). Collectively, the findings revealed that the knockdown of circ-DMNT1 promoted HTR-8/SVneo cell viability, proliferation, migration, and invasion, induced cell cycle progression, but inhibited apoptosis.

### Upregulation of circ-DMNT1 inhibited trophoblast cell survival

**Fig. 3:**
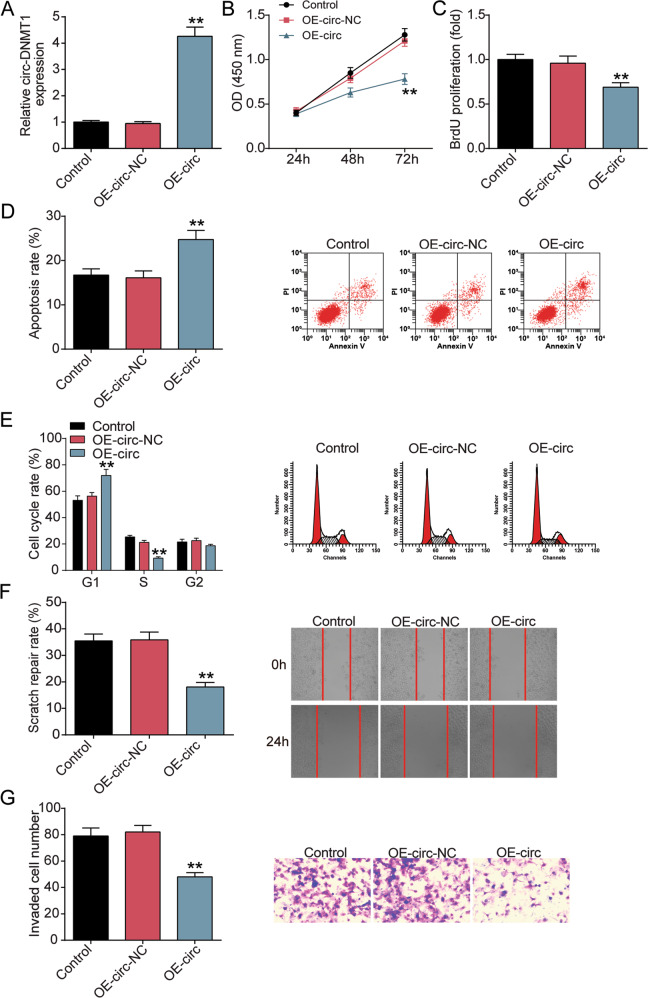
Upregulation of circ-DMNT1 inhibits trophoblast cell survival. **A** HTR-8/SVneo cells were transiently transfected with circ-DMNT1 pcDNA3.1 (OE-circ) for 48 h. qRT-PCR analysis upregulated expression of circ-DMNT1. **B** CCK-8 assays were implemented to detect the viability of OE-circ-treated HTR-8/SVneo cells. **C** BrdU assays were implemented to detect the proliferation of OE-circ-treated HTR-8/SVneo cells. **D** The apoptotic rates of HTR-8/SVne cells transfected with OE-circ were measured by flow cytometry. **E** The cell cycle of HTR-8/SVne cells transfected with OE-circ was measured by flow cytometry. **F** Wound scratch assay was performed to detect the role of circ-DMNT1 on OE-circ-treated HTR-8/SVne cell migration in vitro. **G** Transwell assays were performed to detect the invasion of HTR-8/SVne cells transfected with OE-circ. **P < 0.001 compared with OE-circ-NC. *N* = 3, repetition = 3.

Subsequently, HTR-8/SVneo cells were overexpressed by OE-circ. As shown in Fig. 3A, the expression level of circ-DMNT1 in the OE-circ group was upregulated by about 4-fold. Functional analysis showed that overexpression of circ-DMNT1 reduced cell viability and proliferation levels of HTR-8/SVneo cells by 40% and 25% (Fig. 3B, C). Flow cytometry analysis showed that upregulation of circ-DMNT1 promoted apoptosis and arrested cell cycle progression in HTR-8/SVneo cells (Fig. 3D, E). In addition, Fig. 3F, G displayed that the scratch healing rate was reduced by about 50% and the number of invasions was reduced by about 30% after circ-DMNT1 overexpression. The results suggested that upregulation of circ-DMNT1 suppressed trophoblast cell survival and metastasis.

### Circ-DMNT1 binded to p53 in trophoblast cells

**Fig. 4:**
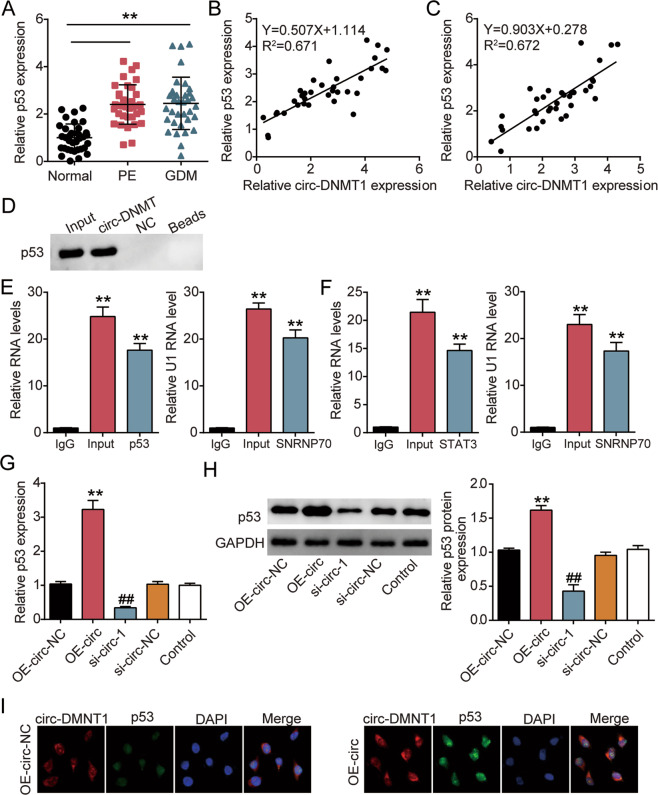
Circ-DMNT1 binded to p53 in trophoblast cells. **A** qRT-PCR analysis of p53 expression in PE (*N* = 35) and GDM placentas (*N* = 35). **P < 0.001. **B** The correlation between circ-DMNT1 and p53 in PE placentas was analyzed by Pearson analysis. **C** The correlation between circ-DMNT1 and p53 in GDM placentas was analyzed by Pearson analysis. **D** The interaction between circ-DMNT1 and p53 was detected by RNA pull-down assay. **E** RIP with rabbit monoclonal anti-p53 and preimmune IgG from HTR-8/SVneo cell extracts. RNA levels in immunoprecipitates were determined by qPCR. Expression levels of circ-DMNT1 RNA are presented as fold-enrichment in p53 relative to IgG immunoprecipitates; U1 binding with SNRNP70 is a positive control. **P < 0.001 compared with IgG. **F** RIP with rabbit monoclonal anti-p53 and preimmune IgG from HTR-8/SVneo cell extracts. RNA levels in immunoprecipitates were determined by qPCR. Expression levels of STST3 RNA were presented as fold enrichment in p53 relative to IgG immunoprecipitates, U1 binding with SNRNP70 is a positive control. **P < 0.001 compared with IgG. **G** Expression of p53 mRNA was downregulated by the si-circ-1 but was upregulated by the OE-circ in HTR-8/SVneo cells. ^##^*P* < 0.001 compared with si-circ-NC. **P < 0.001 compared with OE-circ-NC. **H** Expression of p53 protein was upregulated by the si-circ-1 but was downregulated by the OE-circ in HTR-8/SVneo cells. **P < 0.001 compared with OE-circ-NC. ^##^*P* < 0.001 compared with si-circ-NC. **I** The localization and expression of p53 (green) in HTR-8/SVneo cells overexpressed circ-DMNT1 (red) were detected by IF and FISH. DAPI was used for nuclear staining. *N* = 3, repetition = 3.

In Fig. 4A, the data showed that, compared with the normal group, p53 expression was upregulated in both PE and GDM. In addition, Pearson analysis showed that the circ-DMNT1 was negatively correlated with p53 in PE and GDM (Fig. 4B, C). In HTR-8/SVneo cells, the interaction between circ-DMNT1 and p53 was explored by RNA pull-down assay, and the expression of p53 in circ-DMNT1 pull-down complex was determined by western blot analysis. It showed that p53 was widely aggregated in the pull-down complex of circ-DMNT1 compared with NC (Fig. 4D). Moreover, RIP detection was performed to verify the binding relationship between circ-DMNT1 and p53 using U1 binding SNRNP70 as a positive control, and it was found that circ-DMNT1 directly bound p53 in HTR-8/SVneo cells (Fig. 4E). It has been reported that p53 interacts with STAT3 mRNA and regulates its expression [20]. Therefore, another RIP was carried out, and the data showed that p53 was combined directly with STAT3 (Fig. 4F). Additionally, qRT-PCR and western blot showed that the interference of circ-DMNT1 inhibited the expression of p53, while the upregulation of circ-DMNT1 increased the level of p53 (Fig. 4G, H). Moreover, immunofluorescence and FISH were used to determine the localization and expression of p53 following circ-DMNT1 overexpression. The results showed that p53 mainly existed in the nucleus in the OE-NC group with weak immunoreactivity, while p53 was distributed in the nucleus and cytoplasm in the OE-circ group with stronger immunoreactivity (Fig. 4I). These data suggested that circ-DMNT1 bound to p53 and upregulated its expression level.

### Abnormal p53 expression affects trophoblast cell survival

**Fig. 5:**
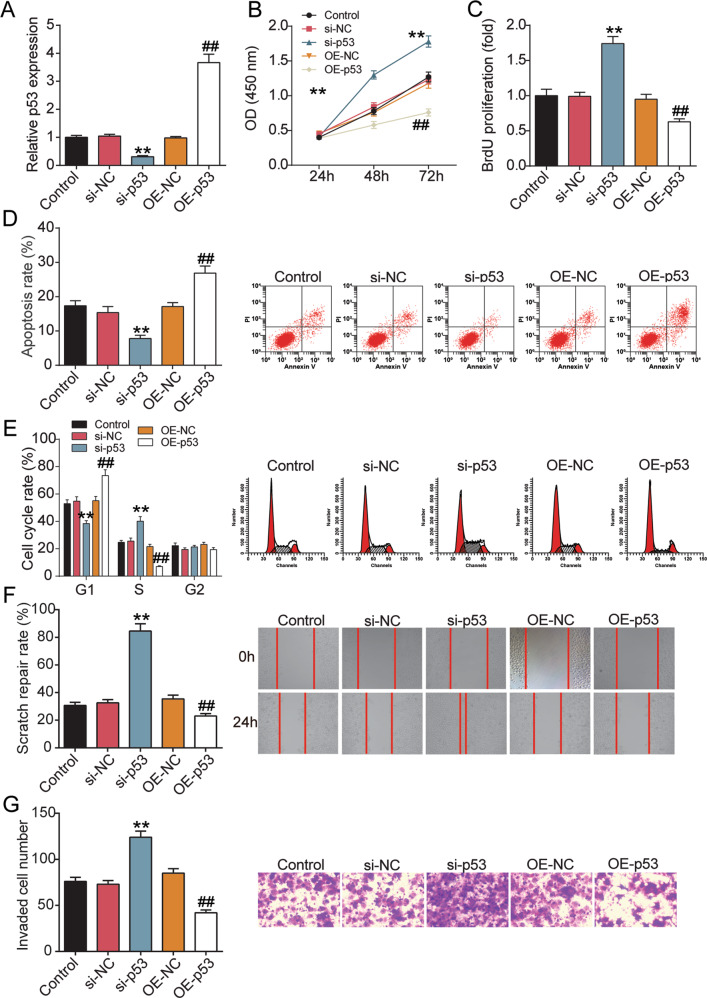
Abnormal p53 expression affects trophoblast cell survival. **A** HTR-8/SVneo cells were transiently transfected with p53 pcDNA3.1 (OE-p53) or siRNA (si-p53) for 48 h. qRT-PCR analysis upregulated expression of p53. **B** CCK-8 assays were implemented to detect the viability of OE-p53 or si-p53-treated HTR-8/SVneo cells. **C** BrdU assays were implemented to detect the proliferation of OE-p53- or si-p53-treated HTR-8/SVneo cells. **D** The apoptotic rates of HTR-8/SVne cells transfected with OE-p53 or si-p53 were measured by flow cytometry. **E** The cell cycle of HTR-8/SVne cells transfected with OE-p53 or si-p53 was measured by flow cytometry. **F** Wound scratch assay was performed to detect the role of OE-p53- or si-p53-treated HTR-8/SVne cell migration in vitro. **G** Transwell assays were performed to detect the invasion of HTR-8/SVne cells transfected with OE-p53 or si-p53. **P < 0.001 compared with si-NC. ^##^*P* < 0.001 compared with OE-NC. *N* = 3, repetition = 3.

In addition, the effect of p53 on the behavior of trophoblast cells was further studied. After transfection, the expression of p53 decreased by 80% in the si-p53 group, while it was increased by about 3.5-fold in the OE-p53 group (Fig. 5A). CCK-8 and BrdU assays showed that knockdown of p53 increased cell viability and proliferation, while overexpression of p53 reduced cell viability and proliferation (Fig. 5B, C). Furthermore, flow cytometry showed that si-p53 transfection reduced apoptosis and promoted cell cycle progression in HTR-8/SVneo cells, whereas OE-p53 treatment promoted apoptosis and cell cycle arrest (Fig. 5D, E). The wound healing experiment displayed that the scratch healing rate in si-p53 cells was increased, while that in OE-p53 cells was decreased (Fig. 5F). The invasiveness of HTR-8/SVneo cells was assessed by matrix gel method and found that increased p53 expression inhibited invasion of HTR-8/SVneo cells, whereas reduced p53 expression promoted invasion (Fig. 5G).

### Circ-DMNT1/p53 activated the JAK/STAT3 signaling pathway in trophoblast cells

**Fig. 6:**
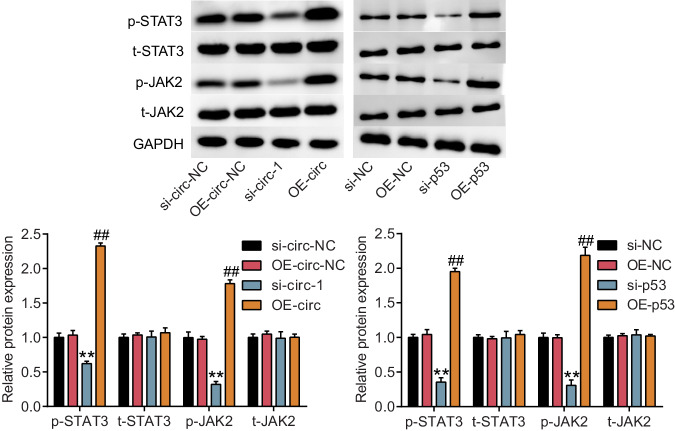
Circ-DMNT1/p53 activates the JAK/STAT3 signaling pathway in trophoblast cells. The expression of JAK2 and STAT3 protein were upregulated by the OE-circ or OE-p53, while were downregulated by the si-circ-1 or si-p53 in HTR-8/SVneo cells. **P < 0.001 compared with si-circ-NC or si-NC. ^##^*P* < 0.001 compared with OE-circ-NC or OE-NC. *N* = 3, repetition = 3.

Furthermore, the regulatory effects of circ-DMNT1 and p53 on JAK/STAT3 signaling pathway-related proteins, including JAK2 and STAT3 were determined. Western blot analysis in Fig. 6 revealed that low expression of circ-DMNT1 or p53 reduced the expression of p-STAT3 by about 30% and 75%, and that of p-JAK2 decreased by about 60% and 65%, respectively. In addition, overexpression of circ-DMNT1 or p53 showed an approximately 2.3- and 1.9-fold increase in p-STAT3 protein level, and about 1.7- and 2.2-fold enhancement in p-JAK2 protein level. In summary, circ-DMNT1 directly bound to p53 and activated the JAK/STAT3 signaling pathway after transcription.

## Discussion

The biological function of trophoblast cells is the key to the establishment, maintenance, and eventual timely termination of physiological pregnancy. It has become a hot spot in the research on the pathogenesis of pregnancy diseases [21]. This study showed that the level of circ-DNMT1 in placental tissue of PE or GDM patients was significantly higher than that of normal pregnant women. In addition, p53 was bound to circ-DNMT1, and it is also upregulated in both PE and GDM. We found that the overexpression of circ-DNMT1 inhibited the viability, migration, and invasion of placental trophoblast cells, induced cell apoptosis and cycle arrest, while the inhibition of circ-DNMT1 expression blocked these processes. Moreover, circ-DNMT1 regulated HTR-8/SVneo cells behavior by binding p53 and activating the JAK/STAT signaling pathway.

The abnormal expression of circRNA is closely related to the occurrence and development of diabetes, cardiovascular disease, cancer, and other complex diseases [22]. Recent studies have shown that circRNA is involved in the occurrence and development of PE and GDM [9, 10]. For example, circSFXN exacerbated PE by inhibiting trophoblast cell invasion, increasing blood pressure and proteinuria in pregnant rats. Circ_0008285 was involved in the development of GDM and its knockdown inhibited HTR-8/SVneo cell proliferation, invasion, and migration [23]. In consistent with these results, we found that the overexpression of circ-DNMT1 in PE and GDM inhibited HTR-8/SVneo cell survival, migration, and invasion, provoked cell apoptosis and cell cycle arrest. Therefore, this study suggested that circ-DNMT1 regulated the biological functions of HTR-8/SVneo cells and participated in PE and GDM.

CircRNA has been proven to interact with proteins and participate in dynamic processes in different tissues, developmental stages, and physiological conditions [11]. Circ-DNMT1 was mainly distributed in the cytoplasm of HTR-8/SVneo cells. Therefore, we speculated that circ-DNMT1 might regulate the gene expression of its downstream targets at a post-transcriptional level. In this study, we found that p53 was also highly expressed in PE and GDM placentas and it was positively correlated with the expression of circ-DNMT1. Besides, RIP showed that circ-DNMT1 bound to p53 in HTR-8/SVneo cells. This result was consistent with the previous study that circ-DNMT1 combined with p53 through its different regions [24]. In addition, the abnormal expression of circ-DNMT1 positively impacted p53 expression. Therefore, we explored the effect of p53 on the cell behavior of HTR-8/SVneo. It was found that low expression of p53 promoted proliferation, invasion, and migration of HTR-8/SVneo cells, inhibited apoptosis, and induced cycle progression, which was similar to the regulation of circ-DNMT1.

The JAK/STAT signaling pathway is commonly associated with a variety of human diseases, including diabetes and hypertension [25, 26]. In addition, compelling studies have shown that the mechanism of JAK/STAT signaling pathways regulating extracellular trophoblast growth and participating in PE development is related to the expression levels of phosphorylated STAT3 and STAT1 [27]. In this study, the data showed that p53 bound to STAT3 and highly expressed circ-DNMT1 or p53 promoted the phosphorylation of JAK2 and STAT3. These results suggested that JAK/STAT signaling pathway might be involved in PE and GDM through the activation of p53 binding with circ-DNMT1.

Although this study provides additional evidence that supports the activation of JAK/STAT signaling pathways in PE and GDM, circ-DNMT1 may also play a regulatory effect through the ways in addition to JAK/STAT pathways. Besides, the lack of animal experiments makes the results of this study less convincing. In the future, we will focus on the key genes and signals downstream of circ-DNMT1/p53 axis, and investigate the changes of related mechanisms in PE and GDM by establishing a pregnant rat model.

In conclusion, our study suggests that increased circ-DNMT1 and p53 in PE and GDM placentas alters the biological function of trophoblast cells in vitro by activating JAK/STAT signaling pathways, and provides a promising therapeutic strategy for PE and GDM.

## Methods

### Specimen collection

The study was approved by the Medical Ethics Committee of The Affiliated Shuyang Hospital of Xuzhou Medical University and informed consent was obtained from each participant. Thirty-five normal pregnant women, 35 women with GDM, and 35 women with PE who underwent cesarean section were included in the study. Inclusion criteria: good health before pregnancy and no history of induced abortion. Exclusion criteria: intrauterine fetal death, any other confounding pathologies, fetal congenital anomalies, hemostatic abnormalities, twin or multiple pregnancies. The placenta was extracted immediately after delivery and washed with sterile PBS, then frozen in liquid nitrogen and stored at −80 °C. All clinical investigations are conducted in accordance with the principles expressed in the Declaration of Helsinki.

### Cell culture and transfection

Human trophoblast cells HTR-8/SVneo purchased from ATCC (USA) were maintained on RPMI-1640 medium containing 10% FBS and 1%P/S (Gibco, USA). All cells were authenticated by short tandem repeat analysis and remained negative for mycoplasma contamination throughout all experiments. The cells were cultured at 37 °C in an incubator containing 5% CO_2_. Lipofectamine 3000 (USA) was used for transfection when the cell confluence reached about 80%. SiRNA and pcDNA3.1 plasmids targeting circRNA DMNT1(si-circ-1/si-circ-2 and OE-circ), targeting p53 (si-p53 and OE-p53) and their negative controls were all from Invitrogen (USA). HTR-8/SVneo cells were collected after transfection for 48 h with 75 nM siRNA and 2 μg/mL pcDNA3.1 for further study.

### qRT-PCR assay

Total RNA was extracted using an RNA isolation kit (Takara, Japan), and then reversed with the PrimeScript RT reagent Kit (TaKaRa, Japan). qRT-PCR was performed on the FastStart Universal SYBR-Green Master Mixes (Roche Diagnostics, USA) per the manufacturer’s instructions on a Q5 thermal cycler (Bio-Rad, USA). GAPDH was used as an internal control for normalization. The relative expression of each gene was determined by 2^−ΔΔCT^. The primer sequences are listed in Table 1.

**Table 1:** The primers used in this study.

Gene	Primer sequence
circ-DMNT1	Forward: 5′-CCACTGTATGAGTGGAAATTAAGA-3′
	Reverse: 5′-AGGGTTTCTTCGGTCGTCAT-3′
p53	Forward: 5’-TGCGTGTGGAGTATTTGGATG-3’
	Reverse: 5′-TGGTACAGTCAGAGCCAACCTC-3’
GAPDH	Forward: 5′-GGAGCGAGATCCCTCCAAAAT-3′
	Reverse: 5′-GGCTGTTGTCATACTTCTCATGG-3′

### Subcellular localization

RNA was isolated from the nucleus and cytoplasm of HTR-8/SVneo cells using PARIS Nuclear/cytoplasmic separation kit (Life Technologies, USA). In short, cells are lysed in a fractionation buffer and centrifuged to separate the nucleus and cytoplasm. The supernatant was then collected in an RNase-free test tube and the remaining sediment was incubated with a cell division buffer to lyse the nucleus and centrifuge the lysate components. The RNA in the nucleus and cytoplasm was eluted, and the subcellular localization of circRNA DMNT1 was detected by qRT-PCR. U6 was used as the positive control in the nuclear part, and GAPDH was used as the positive control in the cytoplasm.

### RNase R treatment

RNase R (4 U/μg; Epicentre Biotechnologies, USA) was added to the RNA of prepared HTR-8/SVneo cells and incubated at 37 °C for 30 min. The RNEasy Minelute Cleanup Kit (Qiagen, USA) was used to purify the incubated RNA, and then the stability of circ-DMNT1 and its linear transcript (linear-DNMT1) was detected by qRT-PCR.

### Immunofluorescent staining and RNA in situ hybridization

HTR-8/SVneo cells from OE-circ and OE-NC groups were washed with cold PBS, then fixed with iced ethanol, followed by appropriate infiltration and blocking steps. Cells were then probed using rabbit anti-p53 (ab90363; Abcam, UK) followed by secondary goat anti-Rabbit IgG H&L (ab150077; Abcam). DAPI was utilized for nuclear staining. Images of the cells were captured under a fluorescence microscope (Olympus, Japan).

Fluorescent in situ hybridization (FISH) was performed based on the protocols of fluorescence in situ hybridization kit (RiboBio, China) using a circ-DMNT1 probe. Briefly, after fixation and permeabilization, cells were incubated with 200 µL of prehybridization solution for 30 min at 37 °C, prior to incubating with circ-DMNT1 probe/hybridization buffer (50% formamide in 2× SSC) overnight. Cells were then washed and imaged as described above.

### CCK-8 assay

Based on the specification, the viability of HTR-8/SVneo cells was detected using the CCK-8 kit (Dojindo, Japan). Briefly, HTR-8/SVneo cells during logarithmic growth were collected and inoculated into a 96-well plate (100 μL cell/well) with a regulatory cell density of 1 × 10^4^ cells/mL. After 24, 48, and 72 h, 10 μL CCK-8 solution was injected into a 96-well culture plate and incubated at 37 °C for 4 h. The absorbance at 450 nm was determined by Microplate Reader (Bio-Rad).

### BrdU assay

Detection of BrdU incorporation into HTR-8/SVneo cells to evaluate cell proliferation based on BrdU incorporation assay (Abcam). The cells were placed on 96-well plates (5 × 10^3^ cells per well) and incubated for 24 h, then 10 M BrdU was added for another 15 h. The cells were then immobilized for 30 min and incubated with 100 μL of BrdU for 60 min. After incubating with 100 μL IgG for 30 min, 100 μL TMB substrate was added and incubated in darkness at room temperature for 30 min. Cell proliferation was evaluated at 450 nm using a microplate reader.

### Flow cytometry assay

For apoptosis, HTR-8/SVneo cells transfected for 48 h were washed with PBS and apoptosis level was detected by FITC-Annexin V/PI detection kit solution (Beijing Biosea Biotechnology, China). Briefly, cells were collected and suspended in binding buffer, then stained with 5 μL FITC-AnnexinV and PI in the presence of 50 μg/mL RNase A, and incubated in darkness for 1 h at room temperature. The percentage of apoptotic cells was detected by FACScan (Beckman Coulter,USA). For Cell cycle, transfected HTR-8/SVneo cells were immobilized overnight in 70% cold ethanol. Subsequently, cells were incubated with RNase A for 30 min at 37 °C, and incubated in thepresence of 400 μL PI in darkness for 1 h at 4 °C. FACScan was utilized to measure cell cycle.

### Wound healing assay

The HTR-8/SVneo cells were placed on a 6-well plate to form a fused monolayer, and then cells were scratched with a 200 μL sterile pipette tip. PBS was washed and incubated in serum-free RPMI1640 medium for 24 h. Cell images were obtained using an inverted microscope (Nikon, Japan) and scratch healing was evaluated.

### Transwell assay

This analysis was performed in a Transwell chamber coated with matrix gel (BD Biosciences, USA). A serum-free medium containing 1 × 10^5^ HTR-8/SVneo cells was added to the transwell upper chamber, and the lower chamber was filled with 600 μL RPMI1640 medium containing 10% FBS. After 24 h of culture, the Transwell chamber was removed. Cells that did not cross the membrane were removed with wet cotton swabs, and the invaded cells were fixed with methanol for 30 s, and then stained with crystal violet for 1 min. The number of cells in three random fields was counted under a microscope.

### Western blot analysis

After transfection for 72 h, the total protein of cells lysed with RIPA lysis buffer (Beyotime, China) was determined by the BCA detection kit (Beyotime). Next, protein (30 µg) was isolated via 10% SDS-PAGE and transferred to PVDF membrane (Millipore, USA), and then blocked with 5% skimmed milk at 25 °C for 2 h. After removal, membranes were incubated with primary antibody: anti-p53 (ab241566; Abcam), p-STAT3 (ab76351; Abcam), STAT3 (ab68153; Abcam), p-JAK2 (ab32101; Abcam), JAK2 (ab39636; Abcam), GAPDH (ab8245; Abcam) at 4 °C overnight. After washing, the membrane was incubated with HRP-labeled secondary antibody 25 °C for 1 h, and then the signal strength of the proteins was analyzed using the ECL detection system (Millipore, USA) and Quantity One software (Bio-Rad, USA).

### RNA immunoprecipitation (RIP)

RIP was detected using the Magna RIP RNA-binding protein immunoprecipitation Kit (Millipore). In simple terms, Cell lysates were prepared with RIP lysates buffer, and the collected lysates were immunoprecipitated with indicated anti-p53 and anti-IgG antibodies (Cell Signaling Technology, USA). RNA was purified from the immunoprecipitation, and the precipitate was detected by qRT-PCR. The interaction of U1 with SNRNP70 was used as a positive control.

### RNA pull-down assay

Biotin-labeled circ-DNMT1 probes were provided by Invitrogen, and RNA pull-down assay was performed according to the instruction of the Magnetic RNA-Protein Pull-Down Kit (Thermo Fisher Scientific, USA). In short, HTR-8/SVneo cell protein extracts were incubated with 2 μg probe and 40 μL streptavidin beads at 4 °C for 2 h, respectively. After centrifugation, beads were collected, RNA binding protein complexes were washed and analyzed by western blot.

### Statistical analysis

Data from the experiment repeated at least three times are presented as mean ± SD. SPSS 19.0 software (SPSS, USA) was applied for statistical analysis. Student’s *t* test and one-way analysis of variance were performed for statistical comparison. To determine whether the data are normally distributed, Kolmogorov–Smirnov test were used. *P < 0.05 indicated that the difference was statistically significant.

## Supplementary information

author agreement

The original blots
