# Correction: The possible involvement of circRNA DMNT1/p53/JAK/STAT in gestational diabetes mellitus and preeclampsia

**DOI:** 10.1038/s41420-024-02065-5

**Published:** 2024-11-04

**Authors:** Dongqin Bao, Chaohui Zhuang, Yan Jiao, Li Yang

**Affiliations:** Center for Reproductive Medicine, The Affiliated Shuyang Hospital of Xuzhou Medical University, Suqian City, Jiangsu Province China

**Keywords:** Cell biology, Diseases

Correction to: *Cell Death Discovery* 10.1038/s41420-022-00913-w, published online 16 March 2022

The original version of this article contained errors.

The authors found that the “Flow cytometry assay” was incorrectly described in the “Materials and Methods” section of the written article. In detail, “Apoptosis assay HTR-8/SVneo cells transfected for 48 h were washed with PBS and apoptosis level was detected by FITC-Annexin V/PI detection kit solution (Beijing Biosea Biotechnology, China). Briefly, cells were immobilized overnight in 70% cold ethanol, then stained with 5 μL PI/FITC-Annexin V in the presence of 50 μg/mL RNase A, and incubated in darkness for 1 h at room temperature. The percentage of apoptotic cells was detected by FACScan (Beckman Coulter, USA).” has been changed to “Flow cytometry assay For apoptosis, HTR-8/SVneo cells transfected for 48 h were washed with PBS and apoptosis level was detected by FITC-Annexin V/PI detection kit solution (Beijing Biosea Biotechnology, China). Briefly, cells were collected and suspended in binding buffer, then stained with 5 μL FITC-Annexin V and PI in the presence of 50 μg/mL RNase A, and incubated in darkness for 1 h at room temperature. The percentage of apoptotic cells was detected by FACScan (Beckman Coulter, USA). For Cell cycle, transfected HTR-8/SVneo cells were immobilized overnight in 70% cold ethanol. Subsequently, cells were incubated with RNase A for 30 min at 37 °C, and incubated in the presence of 400 μL PI in darkness for 1 h at 4 °C. FACScan was utilized to measure cell cycle.”
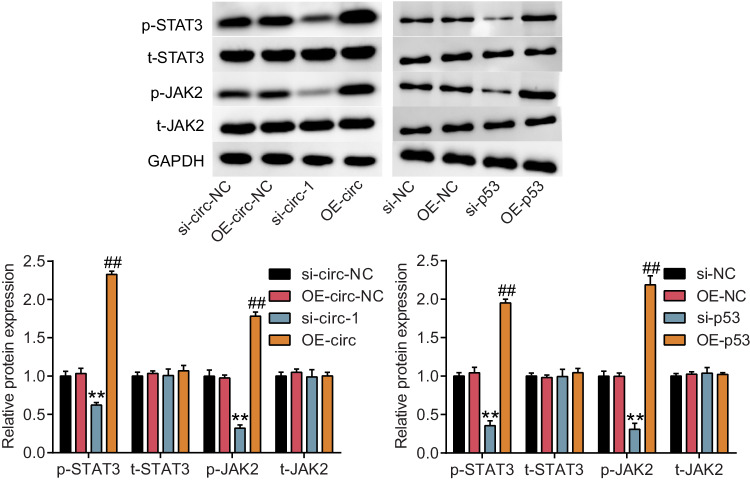

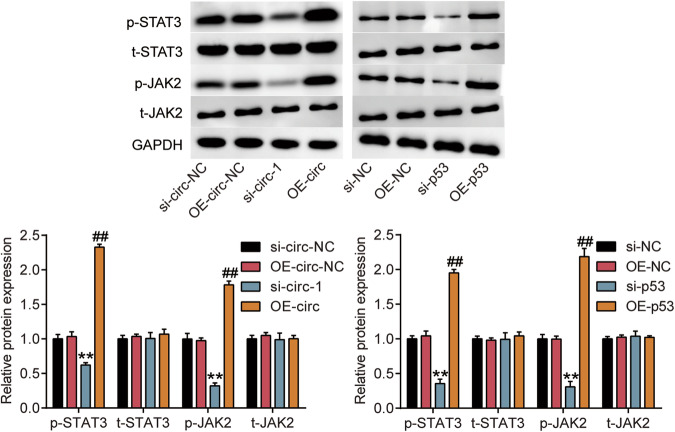


In addition, an incorrect western blot band was entered for t-JAK2 (the left) in Fig. 6. And, the western blot figure above is the corrected Fig. 6, while the one below is incorrect. The authors apologize for these errors.

The original article has been corrected.

