# Supplementary figures and images for: The possible involvement of circRNA DMNT1/p53/JAK/STAT in gestational diabetes mellitus and preeclampsia
Source: Cell Death Discov. 2022 Mar 16;8:121. doi: 10.1038/s41420-022-00913-w (PMC8927128; doi:10.1038/s41420-022-00913-w)

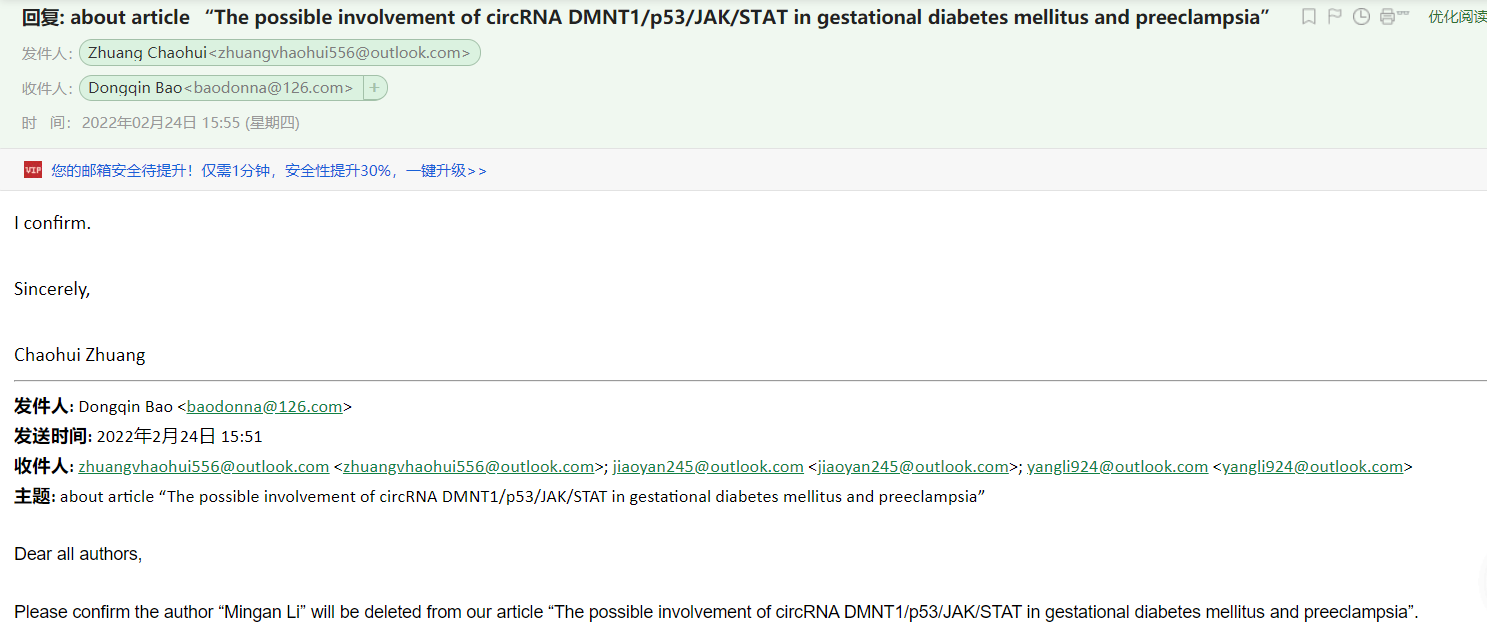

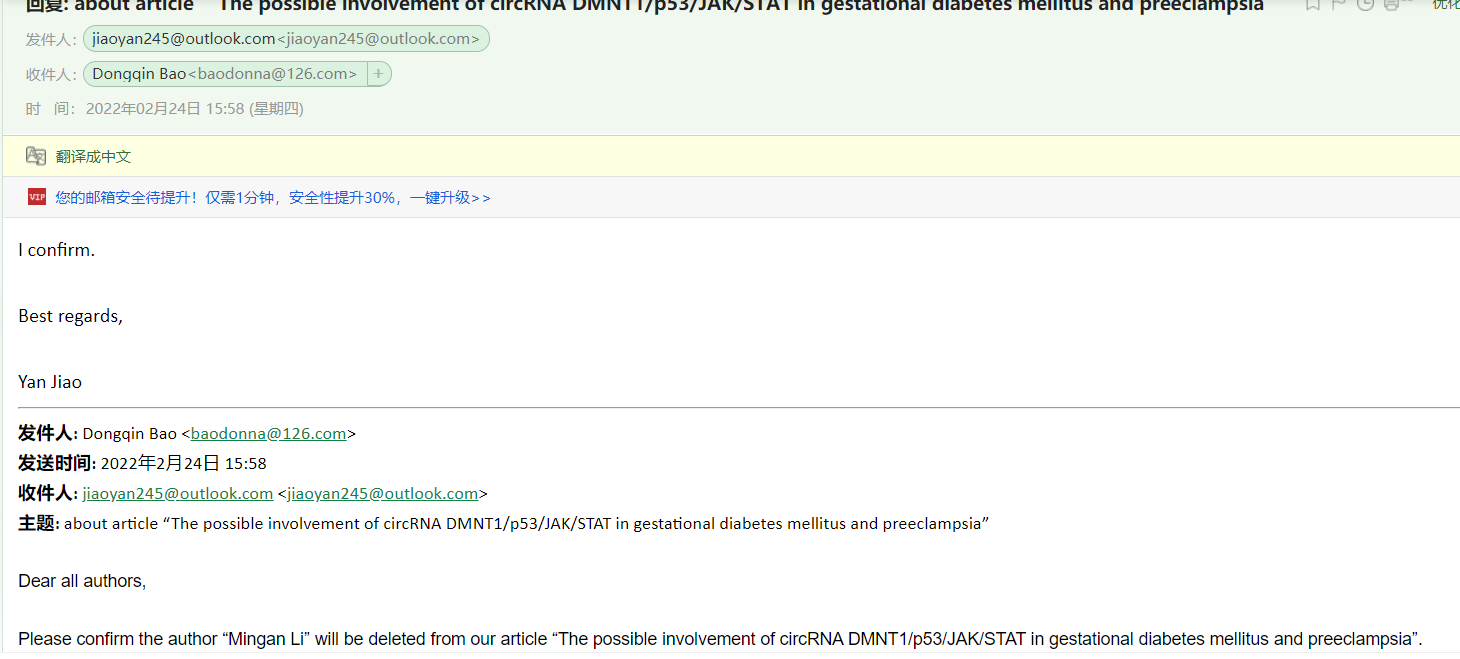

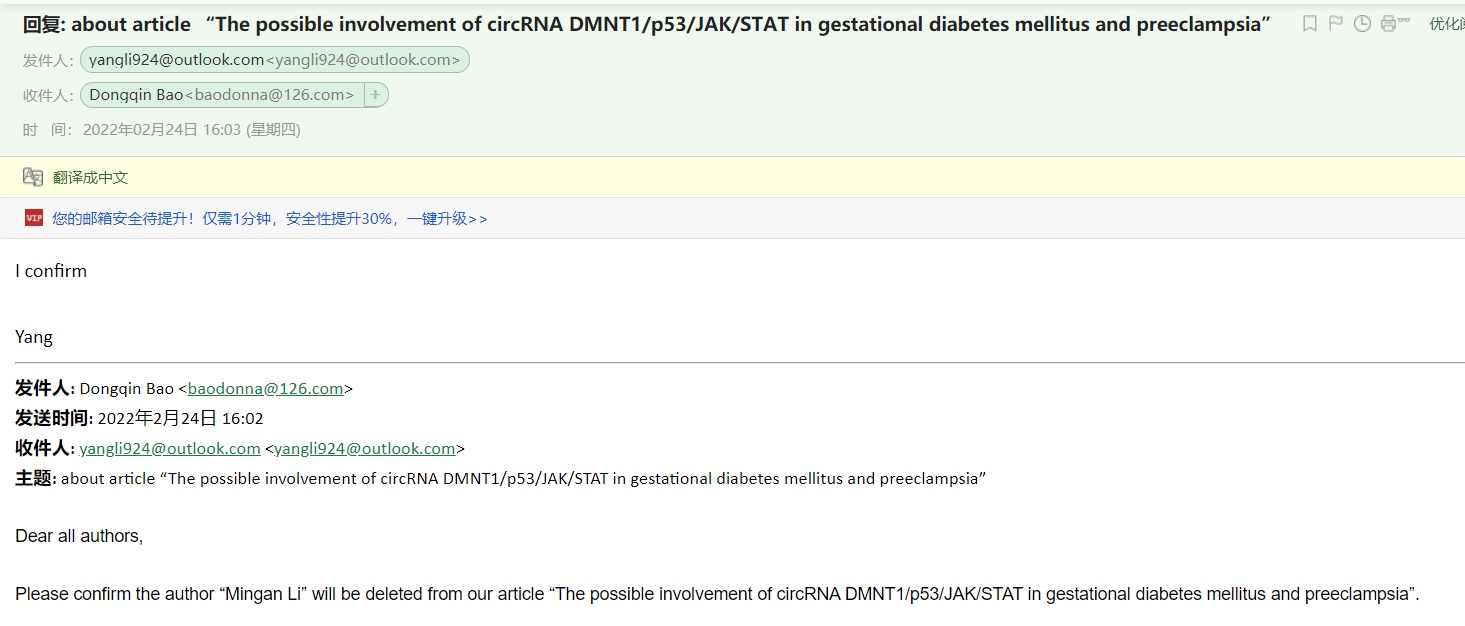

Supplement: Supplementary file 1 — author agreement [file 41420_2022_913_MOESM1_ESM.docx]

The original blots


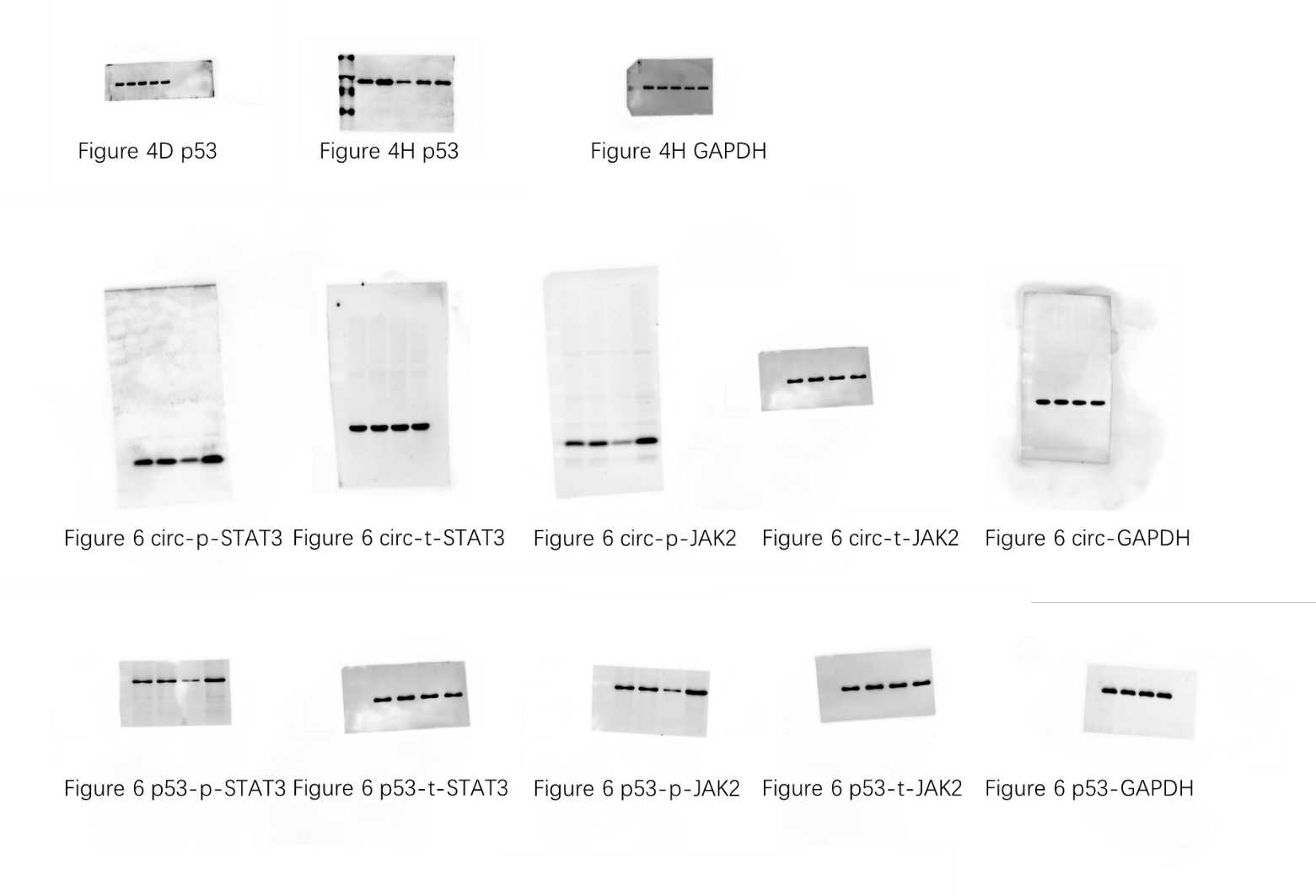

Supplement: Supplementary file 2 — The original blots [file 41420_2022_913_MOESM2_ESM.docx]
